# A quality improvement project (QIP) to address communication and safety concerns from the on-call team at the Bethlem Royal psychiatric hospital out-of-hours through the introduction of weekend safety huddles

**DOI:** 10.1192/bjo.2021.470

**Published:** 2021-06-18

**Authors:** Helen Allis, Mariam Zahedi, Thomas Stephenson, Michael Newson, Anil Kumar

**Affiliations:** South London and Maudsley NHS Foundation Trust

## Abstract

**Aims:**

There have been long-standing concerns about communication and safety on the Bethlem site out-of-hours due to its size, acuity and the number of specialist services; these issues were exacerbated by the COVID-19 pandemic. A Quality Improvement Project was designed to address communication and safety concerns from the on-call team at the Bethlem Royal psychiatric hospital out-of-hours through the introduction of weekend safety huddles.

**Method:**

Daily weekend safety huddles were introduced to improve communication regarding workload, acuity, new admissions, seclusion reviews, deteriorating patients; and to improve team cohesiveness and trainee support out-of-hours.

The QIP team involved the deputy medical director, the associate director for speciality units, consultants, the college tutor, specialty registrars and core psychiatry trainees. Prior to initiating the huddles, the QIP team met to decide which specialties to involve, to agree on an agenda and liaise with other sites regarding existing huddles. Once the huddles began in April 2020, the team met periodically to agree next courses of action and to troubleshoot. The huddles initially involved acute services and eventually included CAMHS, Forensic, Older Adults, Specialist Units, all on-call consultants, the on-call registrar, two core trainees, the psychiatric liaison manager and the duty senior nurse.

**Result:**

Data were gathered throughout the QIP using Likert scale surveys which were sent to all junior doctors on the out-of-hours rota. Paper surveys were used initially but were later replaced with Microsoft Forms to ensure anonymity.

The percentage of respondents who answered “most of the time” or “all of the time” increased across all parameters when comparing data from before and after implementation of the safety huddles.

These results included improvement in: understanding of workload and acuity (9% before vs 69% after), discussion of new admissions on site (4% before vs 90% after), discussion of patients with deteriorating mental health (35% before vs 90% after) and physical health (22% before vs 83% after), understanding of number of patients in seclusion (61% before vs 93% after) and feeling part of a cohesive “on-call” team (17% before vs 86% after). In addition, the results suggested a reduction in frequency of safety concerns on site (83% answered at least “sometimes” before vs 62% after).

**Conclusion:**

The results of the final survey demonstrated a measurable and positive impact on communications between the out-of-hours team, improved team cohesiveness and a reduction in safety concerns. The lessons learnt also influenced decisions made in formatting safety huddles at other trust sites.

